# Efficacy and Safety of Wilac L Probiotic Complex Isolated from Kimchi on the Regulation of Alcohol and Acetaldehyde Metabolism in Humans

**DOI:** 10.3390/foods13203285

**Published:** 2024-10-16

**Authors:** Hwayeon Sun, Sangmin Park, Jiye Mok, Jeonghyun Seo, Nicole Dain Lee, Byungwook Yoo

**Affiliations:** 1 Microbiome R&D Center, Pharmsville, Co., Ltd., Seoul 07793, Republic of Korea; ssunhwayeon@gmail.com (H.S.); plan1@pharmsville.com (S.P.); plan2@pharmsville.com (J.M.); tjwjdguswkd@naver.com (J.S.); 2 School of Medicine, Georgetown University, Washington, DC 20007, USA; ndl37@georgetown.edu; 3 Department of Family Medicine, Soonchunhyang University Seoul Hospital, Seoul 04401, Republic of Korea

**Keywords:** alcohol metabolism, acetaldehyde, alcohol, hangover relief

## Abstract

Alcohol-related hangovers impact both physical and mental wellness, largely due to acetaldehyde levels produced through alcohol metabolism. The present study investigated the efficacy and safety of the Wilac L probiotic complex (*Levilactobacillus brevis* WiKim0168 and *Leuconostoc mesenteroides* WiKim0172 isolated from kimchi) in improving hangovers post-alcohol consumption. This study was conducted as a randomized, double-blind, crossover placebo-controlled clinical trial from August 2023 to February 2024. Subjects (*n* = 26) were randomized into six test groups consuming three products, the Wilac L probiotic complex, Wilac L35 (Wilac L probiotic complex with *Pyrus pyrifolia* Nakai), or placebo, in different orders with crossover after a wash-out interval of 7–10 days. Blood alcohol and acetaldehyde concentrations were measured 0, 0.25, 0.5, 1, 2, 4, 6, and 15 h after alcohol consumption. The blood acetaldehyde levels measured with Wilac L probiotic complex supplementation were significantly lower than the control at 0.25 (*p* = 0.0381), 0.5 (*p* = 0.0498), and 1 h (*p* = 0.0260) post-consumption. The blood acetaldehyde levels after Wilac L35 consumption compared to the control are significant at 0.25 (*p* = 0.0115), 0.5 (*p* = 0.0054), 1 (*p* = 0.0285), 2 (*p* = 0.0113), and 6 h (*p* = 0.0287) post-consumption. No significant adverse events were reported. The Wilac L probiotic complex is associated with decreased blood acetaldehyde levels and improved subjective hangover symptoms.

## 1. Introduction

Alcohol is one of the most commonly abused beverages by modern people. In the case of South Korea, as of 2016, the monthly drinking rate for adults (over the age of 19) was 75.3% for men and 48.9% for women. High-risk drinking rates were 21.1% for men and 6.4% for women, with an annual alcohol consumption of 12.3 L per person [1]. As a result, Korea was ranked 15th among 194 countries according to the WHO, indicating that both the absolute drinking rate and relative alcohol consumption are very high in Korea [2,3,4]. The global average alcohol consumption in 2016 was 6.4 L per person, while in European countries like Germany and France, consumption levels were significantly higher, ranging from 12 to 13 L per person. In the United States, the consumption stood at approximately 9.97 L per person. These statistics highlight the global variation in alcohol consumption and underscore the relatively high rates in South Korea compared to the global average [5,6].

According to the National Health and Nutrition Examination Survey released by the Korean Ministry of Health and Welfare, the annual alcohol consumption in Korea is continuously increasing. Various reasons contribute to this trend as a result of the diversification of modern lifestyles, including increased mental and physical activity and excessive daily stressors. Consequently, the frequency of alcohol intake has increased, and alcohol has become an essential element in social gatherings. In order to maintain health and wellbeing, hangover recovery management is essential [7].

Alcohol ingestion directly contributes to dehydration, electrolyte imbalance, gastrointestinal disturbances, hypoglycemia, circadian rhythm imbalances, and sleep [7]. A hangover refers to the unpleasant physical and mental experiences and decreased physical and mental work capacity that follow alcohol consumption. Major symptoms include physical issues, such as headache, muscle pain, nausea, gastrointestinal problems, sweating, and dizziness, as well as psychological issues, such as depression, lethargy, and anxiety [2].

Various causes of hangovers include dehydration, alcohol toxicity and its related metabolites (acetaldehyde, formaldehyde, acetone, etc.), and nutrient deficiencies from malabsorption including blood sugar, vitamin, and mineral levels. Hangover severity varies greatly among individuals depending on genetic differences and environmental conditions (nutrition, physical activity, degree of dehydration, and overall health).

Research shows that hangovers impact the quality of life by reducing the quality and duration of sleep [8], which leads to higher depression and anxieties tendencies [9]. Thus, it is important to address hangover improvement. When alcohol is ingested, it is metabolized in the body: in the cytoplasm by alcohol dehydrogenase (ADH), in the endoplasmic reticulum by CYP2E1 (Cytochrome P450 2E1), and in peroxisomes by catalase (CAT) into acetaldehyde. It then moves to the mitochondria, where it is further oxidized into acetic acid by non-specific aldehyde dehydrogenase (ALDH) and finally excreted as urine and carbon dioxide (CO_2_). Acetaldehyde is the primary metabolite produced during alcohol oxidation and is a significant factor in hangovers. Acetaldehyde contributes more to the toxic effects of alcohol consumption [10] than alcohol itself and can cause symptoms such as increased pulse, sweating, flushing, nausea, and vomiting when it crosses the blood–brain barrier [11].

Although there have been reports of medications affecting alcohol metabolism to improve hangovers, there are concerns about the safety of these chemically-synthesized substances. Thus, there have been attempts to discover safer natural and food-derived substances for hangover improvement. Probiotics refer to live microorganisms that, when consumed in adequate amounts, provide health benefits to the host [12]. Harmful microorganisms inhabiting the gut, depending on the types and quantities, can cause harmful liver conditions such as cirrhosis, non-alcoholic fatty liver disease, and alcoholic liver disease. In particular, intestinal bacteria, enterococci, and streptococci can increase intestinal permeability and bacterial translocation, leading to high levels of lipopolysaccharides and bacterial substances in the blood, and can cause portal hypertension and liver cell damage as a result. However, there are increasing reports of probiotics effectively restoring gut health [13,14,15].

Current research suggests that the gut–liver axis plays a key role in alcohol metabolism and the resulting hangover symptoms. Alcohol consumption disturbs gut microbiota, increases intestinal permeability (leaky gut), and leads to systemic inflammation, which exacerbates hangover symptoms such as nausea, headaches, and fatigue [16]. Probiotics, such as *Levilactobacillus brevis* and *Leuconostoc mesenteroides*, may help restore gut barrier function, reduce inflammation, and improve the detoxification of alcohol metabolites like acetaldehyde, which is highly toxic [4,16].

Several studies have explored the use of probiotics for improving gut health, showing their potential to restore gut microbiota and enhance gut–liver signaling [4,5]. Bull-Otterson et al. demonstrated how probiotics can mitigate an alcohol-induced liver injury by reducing gut permeability and inflammation [17]. However, while there are increasing reports of probiotics’ benefits in this context, most studies are preclinical, and large-scale human trials are still limited. More investigation is needed to fully understand the specific strains and dosages that are effective for alleviating hangovers and their long-term safety.

Furthermore, individuals carry different variants of ADH and ALDH genes, which encode the major enzymes in alcohol metabolism and can affect their alcohol consumption and risk of alcoholism [18]. ALDH2 is a well-known gene associated with an increased risk of alcoholism. An ALDH2 coding variant protects against alcoholism by producing an inactive ALDH enzyme, leading to the accumulation of acetaldehyde [19]. To accurately assess the effects of acetaldehyde breakdown in research studies, it is crucial to exclude individuals with homozygous variants of the ALDH2 gene.

The *Lactobacillus brevis WiKim0168* and *Leuconostoc mesenteroides WiKim0172* strains in this study were specifically selected as probiotics for their unique properties such as their potential to alleviate hangovers. Previous studies were conducted in vitro and in vivo [20], but their effectiveness in reducing hangover symptoms in humans has not yet been fully explored.

Our study examines a critical gap in the current body of knowledge around the effects of these strains on hangover relief. We aim to explore the health benefits associated with these strains, contributing to the scientific understanding of potential applications for functional foods, probiotic supplements, and therapies targeting alcohol-related symptoms with *Leuconostoc mesenteroides.*

Thus, the present study evaluated the efficacy and safety of the Wilac L probiotic complex (*Levilactobacillus brevis* WiKim0168 and *Leuconostoc mesenteroides* WiKim0172, both isolated from kimchi) in improving hangovers compared to a placebo in individuals who experience hangovers after alcohol consumption.

## 2. Materials and Methods

### 2.1. Subjects

This study was conducted at the Global Medical Research Center from August 2023 to February 2024. The subjects were adults between the ages of 19 and 40 with a body mass index (BMI) between 18.5 kg/m^2^ and 24.9 kg/m^2^, who typically consume one to three bottles of soju per day and experience hangovers after consumption. All subjects voluntarily agreed to participate in the study. Exclusion criteria included subjects homozygous for the ALDH2 gene (Qubit™dsDNA Quantification Assay Kits, Invitrogen Ltd., Carlsbad, CA, USA), patients with peptic ulcers, reflux esophagitis, gastrointestinal diseases (such as Crohn’s disease), or a history of gastrointestinal surgery. Individuals with alcohol-use disorders or addiction, those who had consumed medications or health supplements related to liver function improvement within two weeks prior to the first visit, and those who had consumed alcohol within one week prior to the first visit were also excluded.

This study was approved by the Institutional Review Board of the Global Medical Research Center (IRB No. GIRB-22D28-ML). All participants received detailed explanations about the purpose and contents of the study from the researchers and voluntarily provided written consent.

### 2.2. Experimental Product

The experimental product in this study was the Wilac L probiotic complex, a 3000 mg opaque capsule containing a nonodorous light-yellow powder of Lactobacillus brevis WiKim0168 and *Leuconostoc mesenteroides* WiKim0172. The product was consumed at a dose of 1.0 × 10^9^ CFU/day. The other experimental product was Wilac L35, a 3000 mg combination of the Wilac L probiotic complex (1.0 × 10^9^ CFU/day) with additional brewer’s yeast, Korean pear (*Pyrus pyrifolia* Nakai) concentrate, and oriental raisin tree extract powder consumed with water. The placebo, identical in appearance and form to the experimental product, was composed of 100% maltodextrin. Subjects were instructed to consume three capsules with water in a single dose. The study involved a single intake of the experimental products, Wilac L probiotic complex and Wilac L35, and the placebo, with a crossover intake after a 7–10-day interval.

### 2.3. Study Design

The study was designed as a randomized, double-blind, crossover, placebo-controlled human clinical trial. Participants who voluntarily signed the informed consent for the clinical trial were subjected to demographic and lifestyle surveys and a review of medical history, medication history, and non-drug therapy history. Subjects underwent physical examination to measure vital signs (blood pressure and pulse), height and weight. Clinical pathology tests, pregnancy tests for women of childbearing age, alcohol metabolism gene tests, and drinking habit questionnaires were also completed. Individuals who met the inclusion and exclusion criteria were then randomly assigned as study subjects. Venous blood samples were collected using EDTA-coated tubes to ensure proper sample preservation. The samples were immediately centrifuged at 3000 rpm for 10 min to separate the plasma, which was then stored at −2 °C to 8 °C until further analysis.

For acetaldehyde measurement, gas chromatography–mass spectrometry (GC–MS) was used for its high sensitivity and specificity in detecting alcohol-related metabolites. Additionally, blood biochemical and histological indicators such as ALT and AST were measured using an automated biochemical analyzer.

Testing was conducted by the Global Medical Research Center, adhering to the standard protocols to ensure the reliability and reproducibility of the results. The subjects were assigned to the following groups: ABC group (Visit 2: Wilac L probiotic complex, Visit 3: Wilac L35, Visit 4: Placebo), ACB group (Visit 2: Wilac L probiotic complex, Visit 3: Placebo, Visit 4: Wilac L35), BAC group (Visit 2: Wilac L35, Visit 3: Wilac L probiotic complex, Visit 4: Placebo), BCA group (Visit 2: Wilac L35, Visit 3: Placebo, Visit 4: Wilac L probiotic complex), CAB group (Visit 2: Placebo, Visit 3: Wilac L probiotic complex, Visit 4: Wilac 35), CBA group (Visit 2: Placebo, Visit 3: Wilac L35, Visit 4: Wilac L probiotic complex).

**Figure 1:**
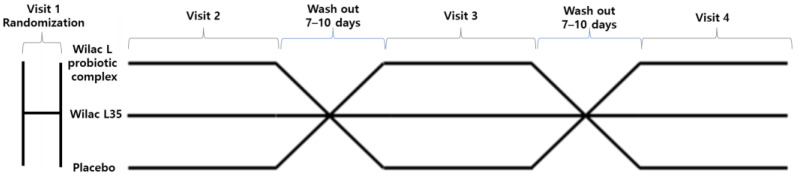
Scheme of the crossover design protocol.

During Visits 2, 3, and 4, the subjects consumed the experimental product (Wilac L probiotic complex, Wilac L35, or placebo) in a single dose. Visit 3 was conducted after a wash-out period of at least 7 days, but no more than 10 days after Visit 2. Visit 4 was similarly conducted after the 7–10-day wash-out period after Visit 3. The ratio of subjects for each group was 1:1:1:1:1 (ABC:ACB: BAC:BCA:CAB:CBA) (Figure 1).

### 2.4. Alcohol Metabolism Gene Test

An alcohol metabolism gene test (ADH1B, ALDH2) was conducted during Visit 1. A 3 mL blood sample was collected and stored at room temperature before analysis by an external laboratory. The samples were immediately discarded after analysis and not used for any secondary purposes.

### 2.5. Safety and Adverse Events

Information about adverse reactions were indirectly explored through non-directive questioning of the subjects during the study period. Additionally, any voluntary reports by participants during hospitalization or confirmed through physical examination, clinical pathology tests, or interviews were noted. Adverse reaction investigations included onset and resolution dates, severity and outcome of adverse reactions, actions taken in relationship to the experimental product, causal relationship of reactions with the product, administered treatment for reactions, and details of the treatment.

### 2.6. Statistical Analysis

Efficacy assessments for this study were analyzed using the Per-Protocol set, and safety evaluation indicators and demographic indicators were analyzed by including all participants who consumed the test food at least once (Full Analysis set). Continuous variables were expressed as mean ± standard deviation (SD) and categorical variables were expressed as *n* (%). All statistical analyses were performed using SAS^®^ (Version 9.4, SAS Institute, Cary, NC, USA) with a significance level set to *p* < 0.05. The statistical significance of the results was evaluated using *p*-values, where *p* < 0.05 was considered statistically significant.

## 3. Results

### 3.1. Demographic Characteristics of the Study Participants

**Figure 2:**
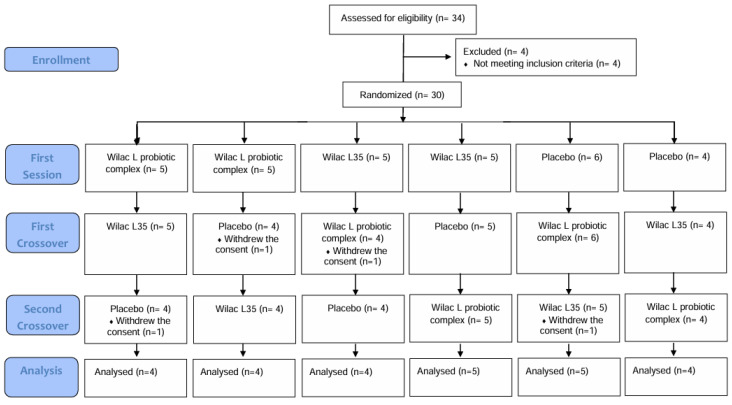
Flowchart of the study.

During the screening process, four participants withdrew their consent and four participants dropped out. Out of the initial thirty-four participants, thirty were randomly assigned to the study. After at least one consumption and efficacy evaluation, twenty-six participants remained registered for the study (Figure 2).

### 3.2. General Participant Characteristics

The demographic information of the FA set study participants is presented in Table 1. The mean age of the participants was 27.88 ± 6.97 years, the mean weight was 64.30 ± 7.62 kg, and the mean BMI was 22.11 ± 1.80 kg/m^2^ (Table 1).

### 3.3. Changes in Blood Acetaldehyde Levels

Changes in blood acetaldehyde levels after alcohol consumption were measured over time (0 h, 0.25 h, 0.5 h, 1 h, 2 h, 4 h, 6 h, 15 h) (Table 2). At 0.25 h after alcohol consumption, blood analysis showed that the mean acetaldehyde level in the Wilac L probiotic complex group was 39.43 ± 23.51 μM while the Placebo group was 53.77 ± 48.90 μM. This demonstrates a statistically significant reduction of 26.7% (*p* = 0.0381) in the Wilac L probiotic complex group compared to the control (Placebo group). Additionally, at 0.5 h post-consumption, the Wilac L probiotic complex group demonstrated a mean acetaldehyde level of 39.76 ± 24.62 μM while the Placebo group was 53.14 ± 47.93 μM, revealing a 25.2% difference (*p* = 0.0498). At 1 h, the Wilac L probiotic complex group’s mean acetaldehyde level was 42.06 ± 30.06 μM and the Placebo group was 57.16 ± 54.28 μM, demonstrating a significant difference of 26.4% (*p* = 0.0260).

At 0.25 h after alcohol consumption, the mean blood acetaldehyde level in the Wilac L35 group was 44.95 ± 32.31 μM and the Placebo group was 53.77 ± 48.90 μM, representing a statistically significant reduction of 16.4% (*p* = 0.0115) in the Wilac L35 group compared to the control (Placebo group). Furthermore, at 0.5 h post-consumption, the Wilac L35 group had a mean level of 44.75 ± 33.14 μM while the Placebo group was 53.14 ± 47.93 μM, demonstrating a 15.8% difference (*p* = 0.0054). At 1 h, the Wilac L35 group had a mean level of 47.75 ± 34.43 μM, while the Placebo group was 57.16 ± 54.28 μM, demonstrating a significant reduction of 16.5% (*p* = 0.0285) between the Wilac L35 group compared to the control. At 2 h, the Wilac L35 group had a mean level of 44.76 ± 32.72 μM and the Placebo group was 55.05 ± 52.55 μM, revealing an 18.7% difference (*p* = 0.0113). At 6 h, the Wilac L35 group had a mean level of 36.62 ± 34.36 μM, while the Placebo group was 52.39 ± 59.64 μM, representing a 30.1% reduction with statistical significance (*p* = 0.0287).

The area under the curve (AUC) for blood acetaldehyde concentration over time was 450.69 ± 363.58 μM·h for the Wilac L35 group and 583.22 ± 618.29 μM·h for the Placebo group. This indicates a statistically significant reduction of 22.7% (*p* = 0.0280) in the Wilac L35 group compared to the control (Placebo group). This reduction suggests that Wilac L35 may mitigate the physiological effects of alcohol consumption and decrease hangover symptoms by modulating blood acetaldehyde levels.

### 3.4. Changes in Blood Alcohol Levels

Changes in blood alcohol levels (%) after alcohol consumption were measured over time (0 h, 0.25 h, 0.5 h, 1 h, 2 h, 4 h, 6 h, 15 h) (Table 3). At every time point, there was no statistically significant difference in the blood alcohol levels between the Wilac L probiotic complex group and Placebo group or between the Wilac L35 group and Placebo group. However, the time to reach the maximum blood alcohol concentration (T_max_) was 1.360 ± 0.550 h in the Wilac L35 group and 1.030 ± 0.551 h in the Placebo group, demonstrating a statistically significant difference between the two groups (*p* = 0.0125). This indicates that although there was no difference in peak blood alcohol levels between the groups at different time points, the time to reach peak blood alcohol levels was prolonged in the Wilac L35 group. This delayed T_max_ suggests that Wilac L35 may affect the kinetics of alcohol absorption, potentially leading to a gradual increase in blood alcohol concentrations, which could affect how hangover symptoms are experienced.

### 3.5. Safety Evaluation

At 15 h post-consumption, there were no statistically significant differences between the groups in all blood biochemical and hematological testing (Table 4). Additionally, no serious adverse or other significant adverse events were observed.

## 4. Discussion

This study conducted a clinical trial on human adults to compare the efficacy of kimchi-derived probiotics in alleviating symptoms and hangovers caused by alcohol consumption. Previous research screened various probiotic strains involving animal models, and among these studies, *Levilactobacillus brevis* WiKim0618 and *Leuconostoc mesenteroides* WiKim0172 were found to be the most effective in alcohol detoxification [20].

Among various probiotic strains, WiKim0168 and WiKim0172 have shown stable growth and resistance to alcohol respectively in cell culture, and the combination of the two strains have demonstrated better ADH and ALDH enzymatic activity together than independently. The same study evaluated the in vivo effects in mice, confirming the alcohol-reducing effects of WiKim0168 and WiKim0172 in Yun et al.’s in vitro cell cultures and our human clinical trials of the Wilac L probiotic complex, made of WiKim0168 and WiKim0172. However, they also found that WiKim0168 and WiKim0172 significantly improved wakefulness from alcohol-induced sleep, a result not observed in our present study [20]. Nevertheless, these observations suggest the Wilac L probiotic complex’s efficacy in improving alcohol metabolism.

Hangovers not only affect individual wellbeing but also have broader social consequences. Symptoms such as headaches, fatigue, and nausea can greatly reduce productivity and increase workplace absenteeism. Moreover, impaired cognitive and motor functions can pose serious risks in scenarios requiring concentration such as driving. The reduced acetaldehyde levels observed in the Wilac L probiotic complex group and Wilac L35 group suggest that probiotics may offer a practical solution for managing hangover symptoms, improving daily function and public safety.

Although our present study did not find a statistically significant difference in the blood alcohol level (%) across time points between the experimental and placebo groups, the T_max_ of the Wilac L35 group was significantly higher than that of the Placebo group, suggesting that alcohol metabolism may occur more rapidly in the Wilac L35 group. Recently, several studies have found that hangovers are more greatly affected by acetaldehyde levels rather than blood alcohol concentration [21]. Furthermore, blood alcohol concentration levels demonstrate a variability more influenced by individual factors (e.g., sex, muscle mass, liver function, and dietary intake) than by hangover relief [10]. Nevertheless, although not statistically significant, the blood alcohol levels in the Wilac L probiotic complex and Wilac L35 groups demonstrate trends of lower levels than the Placebo group, suggesting alcohol metabolism may be faster with consumption of these products.

Recent research has demonstrated, as previously mentioned, that blood acetaldehyde levels are a critical factor in determining the severity of hangovers [21]. Acetaldehyde is a metabolite of alcohol but is more reactive and toxic than alcohol itself and is a primary cause of hangover symptoms. Acetaldehyde remains in the body in large amounts without being oxidized to acetic acid, affecting the autonomic nervous system and causing hangover symptoms such as headache, hyperventilation, drowsiness, vasodilation, tachycardia, and hypotension as a result. Even with an equivalent amount of alcohol consumed, a lower level of acetaldehyde dehydrogenase may result in prolonged hangover symptoms. The current literature has shown that alcohol consumption and sensitivity are associated with genetic variants of ADH and ALDH, enzymes involved in alcohol metabolism and acetaldehyde elimination [22]. Thus, many studies have investigated the correlation between acetaldehyde levels and hangover severity [23,24,25,26,27,28]. The enzyme acetaldehyde dehydrogenase 1 (ALDH-1) is responsible for alcohol metabolism while ALDH-2 eliminates the toxic acetaldehyde produced from alcohol metabolism, alleviating oxidative stress. A common ALDH2 genetic variant among East Asians, including Koreans, affects acetaldehyde levels in the blood and alcohol-associated behavior, increasing the risk for alcoholic cirrhosis [29]. Particularly among East Asians, many individuals have lower levels of ALDH and since genetic variation in ALDH may affect the severity of hangovers [30], subjects homozygous for the ALDH2 gene, identified through alcohol metabolism genetic testing, were excluded from this study.

Acetaldehyde damages cells and DNA, harming liver and brain cells in particular. Therefore, it is important to quickly excrete or eliminate acetaldehyde from the body to alleviate hangovers. In the present study, the Wilac L probiotic complex group showed statistically significant differences in acetaldehyde levels compared to the Placebo group at 0.25, 0.5, and 1 h post-consumption. The Wilac L35 group demonstrated significantly lower levels of acetaldehyde levels compared to the Placebo group at 0.25, 0.5, 1, 2, and 6 h post-consumption. This suggests that the probiotics and Wilac L35 effectively broke down acetaldehyde in the body, resulting in the lower levels seen compared to the control. High levels of acetaldehyde in the body correlate with a greater severity of hangover symptoms. The AUC (area under the plasma level-time curve) represents the degree of drug bioavailability and reflects the total amount of active drug reaching systemic circulation. It is an important indicator in drug development to determine how long and how effectively a drug remains and acts in the body. In our study, the AUC in the Wilac L35 group was significantly lower than the Placebo group, which suggests that less acetaldehyde remained in the body. This further suggests that the Wilac L probiotic complex and Wilac L35 effectively break down acetaldehyde.

Several studies have also demonstrated that probiotics can help activate liver function [14,15,31]. Recently, a meta-analysis based on animal studies demonstrated that probiotic intervention can increase intestinal epithelial barrier (IEB) integrity, alleviate imbalances in intestinal microbiota, and improve liver function, blood lipids, inflammatory factors, and other indicators in ALD mice [32]. Probiotics regulate gut microbiota to decrease liver endotoxin levels and downregulate NF-kB expression, reducing inflammatory marker (IL-6, TNF-α, IFN-γ, etc.) production [33]. Liu et al. demonstrated that probiotics enhance mucus production and expression of tight junction (TJ) proteins, reverse acute alcohol-induced microbiota imbalances, and maintain the integrity of the IEB, thereby reducing liver lipopolysaccharide (LPS) levels [34].

The mechanisms by which probiotics affect alcohol-related liver diseases and other alcohol-related conditions are not clearly identified yet. However, several key underlying mechanisms by which probiotics exert their effects have been described including antioxidant activity, alterations in liver lipid metabolism, downregulation of inflammatory mediators, improvement of IEB function, regulation of the mucosal immune system, and modulation of the gut microbiota [35]. Generally, the most common mechanisms explained are the maintenance of IEB function and modulation of gut microbiota by probiotics [36,37].

In the case of Wilac L35, Korean pears (*Pyrus pyrifolia* Nakai) were used. Lee et al. describe how pears have traditionally been used for hangover alleviation. Korean pears contain higher levels of total sugars, potassium, and water compared to Western pears and are known to mitigate the effects of alcohol and related hangovers [27]. Additionally, they stimulate liver enzymes to increase the rate of alcohol metabolism [38]. Our findings on the efficacy of Wilac L35 can be attributed to the synergistic effects of both the Wilac L probiotic complex and pear extract on relieving hangover symptoms.

Jung et al. conducted a similar crossover study with Duolac ProAP4, containing *Lactobacillus gasseri* CBT LGA1, *Lactobacillus casei* CBT LC5, *Bifidobacterium lactis* CBT BL3, and *Bifidobacterium breve* CBT BR3 on alcohol metabolism. They found that individuals heterozygous for the ALDH2 mutation (ALDH2*2/*1) demonstrated a significant decrease in blood alcohol and acetaldehyde concentrations, indicating faster alcohol metabolism in heterozygous individuals compared to homozygous individual (ALDH2*1/*1) [39]. This supports our findings with the Wilac L probiotic complex significantly decreasing blood acetaldehyde levels, suggesting the effectiveness of probiotics like the Wilac L probiotic complex after alcohol consumption [40].

According to the safety evaluation results, no significant adverse events related to consumption were reported. There were no meaningful changes in vital signs, clinical blood tests, or urinalysis that would impact safety.

Based on the blood ethanol and acetaldehyde levels and AUC results derived from animal experiments, the group that consumed alcohol with hangover-relief functional beverages maintained lower levels of blood alcohol and acetaldehyde and a lower AUC compared to the group that only consumed alcohol. This suggests that these methods are appropriate for evaluating the functionality of hangover relief. Additionally, although the initial blood acetaldehyde levels in the experimental group were higher, these levels decreased over time and after 3 h post-consumption, they were significantly lower than the control. The AUC for acetaldehyde was also lower in the experimental group compared to the control group. These findings suggest that hangover-relief probiotics facilitate the rapid absorption of alcohol and quickly metabolize acetaldehyde, thereby reducing its concentration and improving hangover symptoms.

Although the results are promising, there are several limitations to this study. The sample size was relatively small, and the study duration was limited. Future research should involve a larger and more diverse population and investigate the long-term effects of the Wilac L probiotic complex. When participants were asked about thirst and sleep disturbances after alcohol consumption, those in the Wilac L35 group reported less thirst compared to the placebo group. However, the present study did not directly observe wakefulness or sleep states that may vary among individuals. Future studies should observe these states to compare against ethanol metabolism levels. Additionally, exploring the underlying mechanisms by which these probiotics enhance alcohol metabolism may provide deeper insight into their therapeutic potential. Alcohol is also primarily metabolized through the ADH pathway, so measuring ADH and ALDH activity may lead to more accurate research outcomes. Therefore, future studies should measure the activity of ADH and ALDH and account for potential differences between gender (male and female), wakefulness, or sleep states in their effects.

## 5. Conclusions

This study contributes to the growing evidence supporting the use of probiotics in managing hangover symptoms. The Wilac L probiotic complex, containing *Levilactobacillus brevis* WiKim0618 and *Leuconostoc mesenteroides* WiKim0172, has demonstrated significant efficacy in reducing blood acetaldehyde levels and improving subjective hangover symptoms. The addition of pear extract in Wilac L35 demonstrated enhanced efficacy, with the Wilac L probiotic complex playing a central role in alleviating symptoms. These findings suggest that the Wilac L probiotic complex could be a valuable addition to strategies aimed at alleviating the side effects of alcohol consumption.

## Figures and Tables

**Table 1:** General characteristic of the subjects at baseline (screening).

Variables	Total Subjects (*n* = 26) ^2^
Age (y)	26.15 ± 6.37
Male	18 (69.23)
Female	8 (30.77)
Height (cm)	171.03 ± 7.44
Weight (kg)	65.61 ± 7.15
BMI ^1^ (kg/m^2^)	22.39 ± 1.66
Systolic blood pressure (mm/Hg)	112.69 ± 12.35
Diastolic blood pressure (mm/Hg)	65.85 ± 11.24
Pulse	80.81 ± 11.53
Quantity of Alcohol consumption (g/week)	79.92 ± 27.92

^1^ BMI: Body mass index (Weight (kg)/Height (m)^2^). ^2^ Values are expressed as mean ± SD or number (%).

**Table 2:** Variation in blood acetaldehyde concentration following alcohol challenge test.

		Wilac L Probiotic Complex (*n* = 26)	Wilac L35 (*n* = 26)	Placebo (*n* = 26)	*p*-Value ^(1)^	*p*-Value ^(2)^
Blood acetaldehyde level (μM)	Before ^#^	5.81 ± 5.04	5.27 ± 5.94	6.49 ± 6.21	0.9546	0.8708
	0 h ^$^	42.60 ± 33.53	52.59 ± 50.97	49.49 ± 42.56	0.1434	0.8375
	0.25 h	39.43 ± 23.51	44.95 ± 32.31	53.77 ± 48.90	0.0381 *	0.0115 *
	0.5 h	39.76 ± 24.62	44.75 ± 33.14	53.14 ± 47.93	0.0498 *	0.0054 **
	1 h	42.06 ± 30.06	47.75 ± 34.43	57.16 ± 54.28	0.0260 *	0.0285 *
	2 h	45.05 ± 30.88	44.76 ± 32.72	55.05 ± 52.55	0.2371	0.0113 *
	4 h	38.21 ± 25.03	43.43 ± 32.98	51.62 ± 60.57	0.1406	0.1261
	6 h	36.63 ± 29.71	36.62 ± 34.36	52.39 ± 59.64	0.0764	0.0287 *
	15 h	4.66 ± 4.35	5.53 ± 5.18	5.96 ± 7.64	0.2119	0.8673
C_max_ (mg/dL)		55.00 ± 35.37	60.74 ± 49.30	68.74 ± 62.33	0.1006	0.1692
T_max_ Median (min–max)		1.96 ± 1.91	2.05 ± 2.05	2.05 ± 2.05	0.5854	0.3283
AUC		428.09 ± 304.15	450.69 ± 363.58	583.22 ± 618.29	0.0732	0.0280 *

Values are presented as mean ± SD. Abbreviation: C_max_, maximum plasma concentration; T_max_, time to reach C_max_; AUC, area under the curve. ^(1)^ Analyzed using paired *t*-test (compared between Wilac L probiotic complex and Placebo groups). ^(2)^ Analyzed using paired *t*-test (compared between Wilac L35 and Placebo groups). ^#^, before alcohol intake, ^$^, immediately after alcohol intake. * Significant at *p* < 0.05; ** significant at *p* < 0.01.

**Table 3:** Variation in blood alcohol concentration following alcohol challenge test.

		Wilac L Probiotic Complex (*n* = 26)	Wilac L35 (*n* = 26)	Placebo (*n* = 26)	*p*-Value ^(1)^	*p*-Value ^(2)^
Blood alcohol level (%)	0 h	0.065 ± 0.032	0.074 ± 0.034	0.077 ± 0.041	0.1189	0.6515
	0.25 h	0.091 ± 0.037	0.086 ± 0.034	0.092 ± 0.036	0.9404	0.2434
	0.5 h	0.111 ± 0.044	0.106 ± 0.039	0.113 ± 0.037	0.7154	0.0837
	1 h	0.128 ± 0.028	0.129 ± 0.039	0.127 ± 0.028	0.8489	0.8644
	2 h	0.120 ± 0.024	0.115 ± 0.027	0.118 ± 0.024	0.7075	0.4257
	4 h	0.084 ± 0.024	0.083 ± 0.025	0.081 ± 0.017	0.2831	0.6241
	6 h	0.043 ± 0.022	0.047 ± 0.026	0.043 ± 0.020	0.8928	0.2966
	15 h	0.000 ± 0.000	0.000 ± 0.000	0.000 ± 0.000	-	-
C_max_ (mg/dL)		0.136 ± 0.029	0.134 ± 0.037	0.139 ± 0.028	0.5428	0.2448
T_max_ Median (min-max)		1.090 ± 0.572	1.360 ± 0.550	1.030 ± 0.551	0.6856	0.0125 *
AUC		0.752 ± 0.228	0.761 ± 0.252	0.748 ± 0.190	0.8569	0.6850

Values are presented as mean ± SD. Abbreviation: C_max_, maximum plasma concentration; T_max_, time to reach C_max_; AUC, area under the curve. ^(1)^ Analyzed using paired *t*-test (compared between Wilac L probiotic complex and Placebo groups). ^(2)^ Analyzed using paired *t*-test (compared between Wilac L35 and Placebo groups). * Significant at *p* < 0.05.

**Table 4:** Safety set—blood biochemical and histological testing at 15 h post-consumption for safety evaluation.

	Wilac L Probiotic Complex *n* = 26	Wilac L35 (*n* = 26)	Placebo *n* = 26	*p*-Value ^(1)^	*p*-Value ^(2)^
WBC (10^3^/μL)	7.24 ± 2.08	7.01 ± 1.89	7.25 ± 2.13	0.9870	0.6559
RBC (10^6^/μL)	4.73 ± 0.49	4.75 ± 0.49	4.72 ± 0.48	0.9547	0.7921
Hb (g/dL)	14.25 ± 1.57	14.33 ± 1.40	14.19 ± 1.43	0.8757	0.7013
Hct (%)	43.46 ± 4.15	43.86 ± 4.19	43.47 ± 4.41	0.9942	0.7378
Platelet (10^3^/μL)	280.90 ± 45.06	290.07 ± 53.51	280.67 ± 46.04	0.9850	0.4885
Neutrophil (%)	50.07 ± 11.51	50.55 ± 9.04	50.77 ± 9.54	0.8056	0.9303
Lymphocyte (%)	38.93 ± 10.57	38.65 ± 8.14	38.19 ± 8.62	0.7745	0.8404
Monocyte (%)	7.06 ± 1.68	6.81 ± 1.69	7.09 ± 1.76	0.9804	0.5840
Eosinophil (%)	3.22 ± 2.39	3.27 ± 2.16	3.24 ± 2.21	0.9411	0.8662
Basophil (%)	0.72 ± 0.45	0.73 ± 0.41	0.72 ± 0.31	0.4524	0.7282
AST(GOT) (U/L)	20.52 ± 7.09	20.86 ± 8.67	38.11 ± 8.62	0.9934	0.9462
ALT(GPT) (U/L)	16.41 ± 11.06	16.82 ± 12.74	20.70 ± 20.65	0.8565	0.9933
Total Cholesterol (mg/dL)	176.24 ± 33.07	179.43 ± 32.65	173.37 ± 3.92	0.9281	0.5555
Glucose (mg/dL)	74.86 ± 3.43	74.79 ± 4.56	75.11 ± 3.92	0.8008	0.7348
Total Protein (g/dL)	6.98 ± 0.41	7.02 ± 0.42	6.92 ± 0.34	0.5479	0.3375
BUN (mg/dL)	13.10 ± 1.97	13.11 ± 2.36	12.78 ± 1.93	0.5548	0.5743
Creatinine (mg/dL)	0.83 ± 0.17	0.83 ± 0.17	0.83 ± 0.16	0.9850	0.9951
Uric Acid (mg/dL)	5.93 ± 1.27	6.06 ± 1.26	6.00 ± 1.28	0.8567	0.8435
Ca (mg/dL)	9.23 ± 0.41	9.26 ± 0.32	9.19 ± 0.36	0.6356	0.4360

Values are presented as mean ± SD. Abbreviation: C_max_, maximum plasma concentration; T_max_, time to reach C_max_; AUC, area under the curve. ^(1)^ Analyzed using paired *t*-test (compared between Wilac L probiotic complex and Placebo groups). ^(2)^ Analyzed using paired *t*-test (compared between Wilac L35 and Placebo groups).

## Data Availability

The data presented in this study are available on request from the corresponding author and first author. The data are not publicly available as the first author needs to apply for a doctoral degree.
